# Border Region Surveillance of Malaria Drug Resistance, Northern Burundi, 2023–2024

**DOI:** 10.3201/eid3204.251711

**Published:** 2026-04

**Authors:** Denis Niyomwungere, Pierre Sinarinzi, Emmanuelle Caspar, Lucas Thiebaut, Pierre-Emeric Strubel, Yssimini Nadège Guillène Tibiri, Laurence Ma, Jérôme Rurihafi, Pierre Ndizeyimana, Pierre Hakizimana, Nestor Niyizompa, Adélin Witonze, Vénant Ndayiragije, Floride Ndayisenga, Malachie Ndikumukiza, Olive Niyonkuru, Jean Claude Niyonsaba, Césaire Bangirinama, Jolis Lazare Bigirimana, Nicaise Ntahondi, Landrine Mugisha, Marcelline Nibakire, Néhémie Nzoyikorera, Denis Sinzinkayo, Didier Menard, Joseph Nyandwi

**Affiliations:** Institut National de Santé Publique, Bujumbura, Burundi (D. Niyomwungere, P. Ndizeyimana, P. Hakizimana, J. Nyandwi); Programme National Intégré de Lutte contre le Paludisme, Bujumbura (P. Sinarinzi, J. Rurihafi, N. Niyizompa, A. Witonze, F. Ndayisenga, L. Mugisha, M. Nibakire, N. Nzoyikorera); Malaria Genetics and Resistance Team, Pathogens Host Arthropods Vectors Interactions Unit, University of Strasbourg, Strasbourg, France (E. Caspar, L. Thiebaut, P.-E. Strubel, Y.N.G. Tibiri, D. Menard); Institut de Recherche en Sciences de la Santé, Clinical Research Unit of Nanoro, Nanoro, Burkina Faso (Y.N.G. Tibiri); Biomics Platform, Institut Pasteur, Université Paris Cite, Paris, France (L. Ma); Centre Hospitalo-universitaire de Kamenge, Bujumbura (V. Ndayiragije, M. Ndikumukiza, O. Niyonkuru, C. Bangirinama, J.L. Bigirimana, N. Ntahondi); Université du Burundi, Bujumbura (M. Ndikumukiza, D. Sinzinkayo, J. Nyandwi); Tanganyika Care Hospital, Bujumbura (J.C. Niyonsaba); Malaria Parasite Biology and Vaccines, INSERM Unit 1347, ParasitInnov, Institut Pasteur, Université Paris Cité, Paris (D. Menard); Laboratory of Parasitology and Medical Mycology, CHU Strasbourg, Strasbourg (D. Menard); Institut universitaire de France, Paris (D. Menard)

**Keywords:** malaria, Plasmodium falciparum, antimicrobial resistance, parasites, antimalarial drug resistance, artemisinin partial resistance, pfkelch13, day-3 positivity, sulfadoxine/pyrimethamine, molecular surveillance, Burundi, Great Lakes region

## Abstract

To evaluated artemisinin partial resistance (ART-R) in malaria in Burundi, during December 2023–June 2024, we studied 423 children <5 years of age with uncomplicated *Plasmodium falciparum* malaria in 8 health facilities in the northern part of the country. After artemether/lumefantrine treatment with only the first dose directly observed, 4.5% remained parasitemic on day 3. No *pfkelch13* mutations, validated or candidate markers of ART-R, were detected. However, markers of antifolate and 4-aminoquinoline resistance were widespread: the *dhfr* triple mutant N51I/C59R/S108N was nearly fixed (92%), *dhps* double and triple mutants were common (41% and 47%), and *pfcrt* CVIET, associated with chloroquine and amodiaquine resistance, predominated (84%). Geographic differences occurred in day-3 positivity and haplotype frequencies. Although ART-R markers were absent, delayed parasite clearance and near fixation of multidrug-resistant haplotypes serve as a warning. Strengthened efficacy monitoring and regional molecular surveillance are urgently needed to prevent drug-resistant *P. falciparum* from becoming established in Burundi.

Malaria remains a leading cause of illness and death in low- and middle-income countries, and sub-Saharan Africa bears the greatest burden. In 2024, an estimated 280 million malaria cases and 610,000 deaths occurred globally, of which 94% of cases and 95% of deaths were in sub-Saharan Africa. Children <5 years of age and pregnant women are the most affected groups (*1*,*2*).

To accelerate control, the World Health Organization (WHO) launched the Global Technical Strategy for Malaria 2016–2030, targeting a 90% reduction in incidence and mortality and elimination in 35 countries by 2030 (*3*). Burundi has scaled up core interventions, including artemisinin-based combination therapies (ACTs) since 2003, long-lasting insecticidal nets, indoor residual spraying, and intermittent preventive treatment in pregnancy (IPTp) (*4*–*7*). First-line treatment shifted from artesunate/amodiaquine to artemether/lumefantrine in 2019 (*4*). More recently, perennial malaria chemoprevention (PMC) was introduced in 5 districts, and RTS,S/AS01 vaccine (GSK, https://www.glaxosmithkline.com) was integrated into routine immunization in March 2025, with initial deployment in 25 of the country’s 47 health districts (*5*,*6*). Despite those efforts, malaria transmission remains intense; 4.9 million cases and 809 deaths were reported by the national surveillance system in 2023–2024, including 2.2 million cases among children <5 years of age (*5*,*6*).

The emergence and spread of drug resistance is a looming threat. Artemisinin partial resistance (ART-R), defined by day 3 parasitemia and validated *pfkelch13* mutations, has been confirmed in Rwanda and Tanzania (*8*–*14*). Given porous borders and high population mobility, Burundi faces increased risk for resistant *Plasmodium falciparum* introduction. We conducted a prospective study in northern Burundi using a simplified day 3 follow-up design after artemether/lumefantrine treatment to assess delayed parasite clearance and the key molecular resistance markers, *pfkelch13*, *pfcrt*, *pfmdr1*, *dhfr*, and *dhps*.

## Methods

### Study Design and Population

#### Study Design and Setting

We conducted the study during December 2023–June 2024 in 8 health facilities across 5 districts of northern Burundi, a high *P. falciparum* transmission zone bordering Rwanda (Figure). Sites were located in Kirundo Province (2 facilities in Kirundo, 1 in Vumbi) and Ngozi Province (3 facilities in Buye, 1 in Ngozi, and 1 in Kiremba). This region experiences intense perennial transmission with seasonal peaks during March–June and October–December. According to national surveillance data, parasitemia prevalence in children <5 years of age reaches 79% in Kirundo and 54% in Ngozi (*15*), and clinical incidence approaches 400 cases/1,000 population annually (*5*,*6*,*15*).

**Figure:**
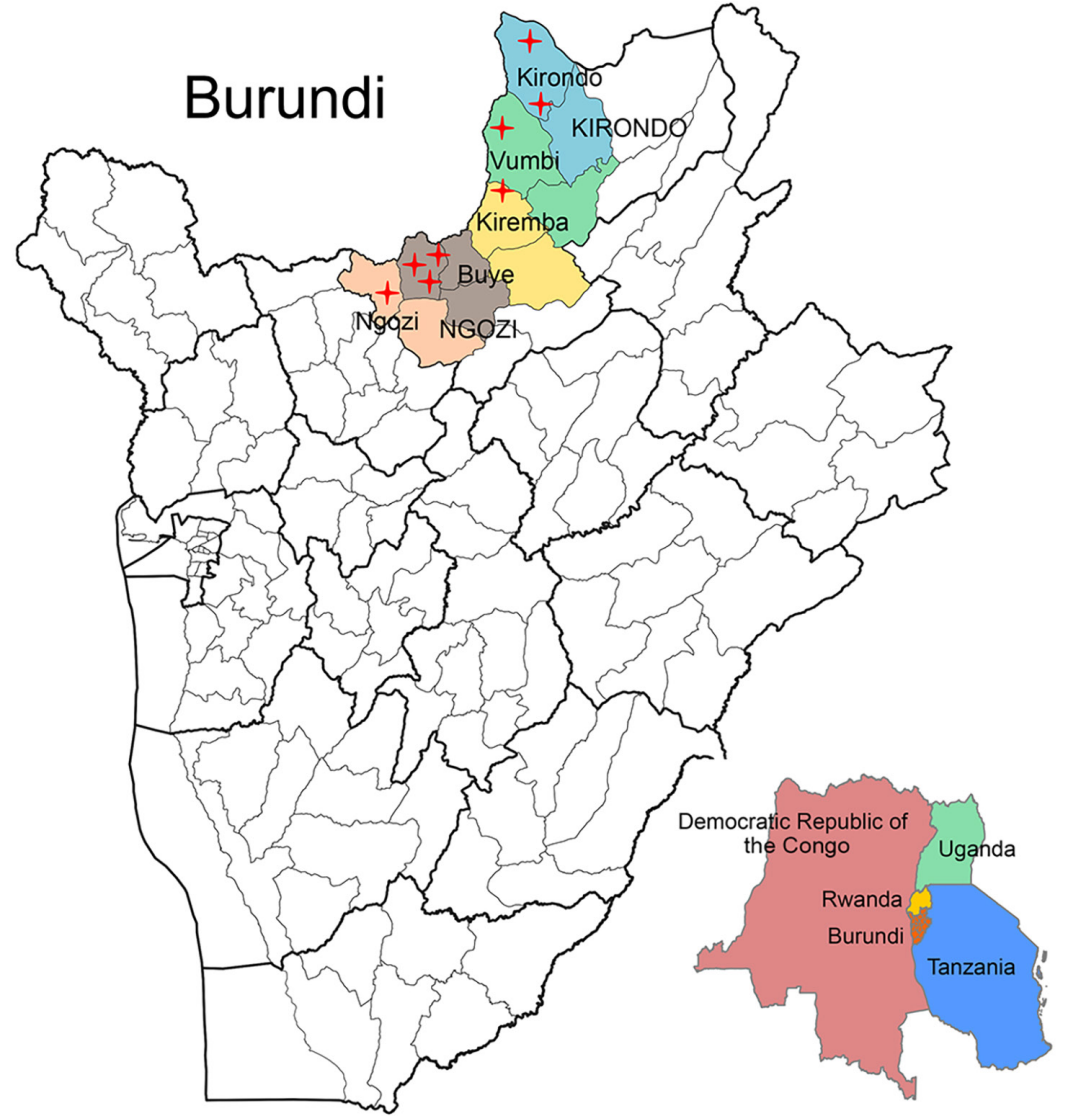
Locations of 8 sentinel health facilities used for study of border region surveillance of malaria drug resistance, northern Burundi, 2023–2024. Red crosses show locations of participating health facilities across the provinces of Ngozi and Kirundo, where clinical and molecular surveillance of artemisinin partial resistance was conducted during December 2023–June 2024. District health facilities included were in Ngozi, Kiremba, and Buye in Ngozi Province and Kirundo and Vumbi in Kirondo Province. Inset shows bordering countries in Africa.

#### Study Population and Eligibility Criteria

We screened children <5 years of age who had fever (≥37.5°C axillary or history of fever within the previous 24 h). Inclusion required microscopically confirmed *P. falciparum* monoinfection. We applied no minimum parasite density or hemoglobin thresholds for enrollment. Exclusion criteria were signs of severe malaria, mixed infections, or known hypersensitivity to artemisinin derivatives.

### Treatment, Follow-up Procedures, and Outcomes

Participants received a standard 3-day course of artemether/lumefantrine (Lumartem DT, https://www.cipla.com; WHO-prequalified dispersible tablets, 20 mg artemether/120 mg lumefantrine) according to Burundi national guidelines (*4*). Dosing followed WHO weight-based recommendations: children 5 to <15 kg received 1 dispersible tablet per dose, and children 15 to <25 kg received 2 tablets per dose. Each treatment course consisted of 6 doses administered at 0, 8, 24, 36, 48, and 60 hours. The first dose was directly observed and was administered with fatty food to enhance lumefantrine absorption. Caregivers were instructed to administer subsequent doses with food at home with reinforced instructions. On day 0 and day 3, we collected capillary blood samples for Giemsa-stained smears and dried blood spots for molecular analysis. On day 3, all children underwent clinical assessment including temperature measurement and symptom evaluation. We referred children with persistent fever or clinical deterioration for management, according to national guidelines. We did not re-treat those with persistent parasitemia but no danger signs because the study assessed day 3 positivity for surveillance purposes rather than to guide individual treatment.

### Microscopy

We prepared thick and thin smears and stained with 10% Giemsa (pH 7.2) for 15 minutes. We used thick smears for parasite detection and density quantification and thin smears for species identification on the basis of parasite morphology when mixed infections were suspected or when species confirmation was required. Two certified microscopists independently read slides at 1,000× magnification; discrepancies >25% in parasite density were resolved by a third reader, and we calculated the final parasite density as the mean of the 2 closest readings. We calculated parasite density per 200 leukocytes (8,000/µL) and recorded gametocyte presence.

### Molecular Analysis

We collected dried blood spots on Whatman 903 filter paper, air-dried, stored with desiccant, and shipped to the University of Strasbourg (Strasbourg, France) for analysis. We extracted DNA from dried blood spots by using the QIAamp DNA Blood Mini Kit (QIAGEN, https://www.qiagen.com). We confirmed *Plasmodium* species by real-time PCR targeting *cytb* and by nested species-specific PCR (*16*).

We analyzed drug-resistance markers by targeted amplicon sequencing of *pfkelch13* (codons 430–720), *pfcrt* (codons 72–76, 93, 97, 145, 218, 343, 350, 353, and 356), *pfmdr1* (codons 86, 184, 1034, 1042, and 1246), *dhfr* (codons 16, 50, 51, 59, 108, and 164), and *dhps* (codons 431, 436, 437, 540, 581, and 613) (*17*). We multiplexed and barcoded amplicons, then pooled, purified, and sequenced on an Illumina MiSeq platform (300-cycle v2 kit; Illumina, https://www.illumina.com). We quality-trimmed reads (Phred score >30; https://www.phrap.com/phred) and aligned reads to the *P. falciparum* 3D7 reference genome (v45) by using CLC Genomics Workbench version 22 (QIAGEN). We called variants that were supported by >20 reads and allele frequency >5%. Reference strains Dd2, 7G8, HB3, and 3D7 served as positive controls (*17*).

### Sample Size Considerations

This surveillance was designed to detect circulating *pfkelch13* mutant parasites, particularly the R561H allele, validated in neighboring Rwanda and Tanzania. We targeted ≈50 children per site (400 total) to ensure broad geographic coverage of the border region while maintaining logistical feasibility. This sample size provides 95% confidence to detect mutant alleles circulating at >0.75% prevalence if none are observed (rule of three: 3/n). The study was not powered for formal interdistrict comparisons; district-level differences in baseline characteristics and molecular markers are reported as exploratory observations to generate hypotheses for future targeted investigations.

### Study Outcomes

The primary outcome was the percentage of children with persistent parasitemia on day 3 (day 3 positivity), defined as microscopically detectable asexual *P. falciparum* parasites on day 3 after treatment initiation. That indicator serves as a clinical proxy for delayed parasite clearance associated with ART-R. Secondary outcomes included the prevalence of molecular markers associated with malaria drug resistance: *pfkelch13* mutations (ART-R), *pfcrt* and *pfmdr1* haplotypes (chloroquine, amodiaquine, and piperaquine resistance), and *dhfr/dhps* mutations (sulfadoxine/pyrimethamine resistance).

### Statistical Analysis

We summarized demographic and clinical variables by using descriptive statistics and compared continuous variables by using the Kruskal-Wallis test and categorical variables by using χ^2^ or Fisher exact tests. We considered p<0.05 significant.

We assessed day 3 positivity after treatment with only the first dose directly observed, estimated prevalence with exact binomial 95% CIs and compared across provinces (Kirundo and Ngozi) to assess geographic heterogeneity. We calculated allele frequencies for *pfkelch13*, *pfcrt*, *pfmdr1*, *dhfr*, and *dhps* and stratified by district. We classified mixed infections as mutant for allele-frequency estimates. We reconstructed inferred *dhfr-dhps* haplotypes from unphased single-nucleotide polymorphism data and compared them between districts; we interpreted results as inferred patterns rather than observed genotypes. We performed analyses with MedCalc version 20.218 (MedCalc Software Ltd, https://www.medcalc.org).

### Ethical Considerations

The study followed the Declaration of Helsinki and WHO guidelines for human research. Approval was obtained from the Burundi National Ethics Committee for the Protection of Human Subjects (approval no. CNE/25/2023). Written informed consent was obtained from parents or legal guardians. Participant confidentiality and data security were strictly maintained.

## Results

We screened 873 febrile children <5 years of age across the 8 health facilities. Of those, 423 met the inclusion criteria and completed clinical follow-up through day 3, corresponding to 1 day after completion of artemether/lumefantrine treatment. Of the 423 samples collected, 365 (86.3%) day 0 samples were successfully confirmed as *P. falciparum* infections and subsequently analyzed by molecular sequencing to address the secondary objective: detection of *pfkelch13* mutations, notably R561H, a validated marker of ART-R. Sequencing success varied by district: 100% in Kiremba and Ngozi, 91% in Kirundo, 75% in Buye, and 76% in Vumbi. We excluded the other 58 samples (13.7%) because of insufficient DNA quality.

The median age of enrolled children was 30 (IQR 17–45) months, and the sex ratio was balanced (1.0). Baseline characteristics showed variation across districts (Table). Median axillary temperatures ranged from 37.8°C (Buye) to 39.0°C (Kirundo). Pretreatment asexual *P. falciparum* parasite density varied widely; median values ranged from 10,543/µL (Vumbi) to 42,400/µL (Kirundo). Gametocyte carriage was detected in 5.4% of children overall, with district-specific prevalence ranging from 1.2% (Kirundo) to 22.0% (Ngozi). Mixed-species infections were uncommon (1.2% *P. malariae* in Ngozi Province), confirming *P. falciparum* as the predominant species.

**Table:** Baseline characteristics and day 3 parasitologic outcomes for patients with *Plasmodium falciparum* malaria, northern Burundi, December 2023–June 2024*

Baseline characteristics	Kirundo Province				Ngozi Province				Total	p value
	Kirundo	Vumbi	Total		Buye	Kiremba	Ngozi	Total		
No. patients	170	82	252		97	30	44	171	423	–
Gender ratio, M/F	0.9	1.5	1.1		0.8	2.0	1.0	1.0	1.0	0.77
Age, mo										
Median	32	30	31		28	29	32	30	30	0.5
95% CI	27–36	22.2–33	27–34.3		23–35.5	17–36.65	28.5–36	25.9–34	28–32	–
IQR	19–47	13.75–38.25	18–46		14–42	15–42	20.75–45	16–42	17–45	–
Weight, kg										
Median	11	10	11		10	11.5	10	10	10	0.5
95% CI	10–12	9–11.2	10–11		10–11	9.2–13.8	10–12.2	10–11	10–11	–
IQR	9–13.5	8.1–12.9	9–13		8–13	10–13	10–14	9–13	9–13	–
Temperature, °C										
Median	39.0	38.3	38.6		37.8	38.1	38.7	38.4	38.5	**0.01**
95% CI	38.6–39.0	38.0–38.5	38.4–38.9		37.8–38.4	37.8–39.0	38.5–38.9	38.0–38.5	38.4–38.6	–
IQR	38.0–39.6	38.0–38.9	38.0–39.2		37.4–38.9	37.7–39.0	38.3–39.0	37.7–39.0	37.8–39.0	–
Pretreatment asexual *P. falciparum* density, parasites/µL										
Median	42,400	10,543	36,734		10,745	12,743	27,661	19,374	27,313	**0.01**
95% CI	33,663–54,525	7,499–39,836	25,325–46,160		3,846–26,436	8,028–38,878	15,887–41,680	10,733–26,650	22,511–35,539	–
IQR	7,566–108,166	2,759–72,886	4,615–102,933		1,625–48,692	5,128–44,000	9,746–59,628	2,400–51,912	3,521–79,513	–
Pretreatment sexual *P. falciparum* density, parasites/µL, %	1.2	3.7	2		7.4	3.3	22.0	10.2	5.3	**0.0003**
Mixed infections with other species, %										
* P. vivax*	0	0	0		0	0	0	0	0	0.08
* P. malariae*	0	0	0		1.1	0	2.4	1.2	0.5	
* P. ovale*	0	0	0		0	0	0	0	0	
Day 3 positivity rate, %†	1.8	13.6	5.7		2.1	0	7.3	3.0	4.5	0.06
Day 3 asexual *P. falciparum* density, parasites/µL										
Median	1,480	240	260		100	–	1,832	103	260	0.96
95% CI	–	177–281	200–1,522		–	–	–	–	175–1,290	–
IQR	5,475–23,172	192–275	202–1,140		98–103	–	494–13,381	85–5681	141–1,659	–
Day 3 sexual *P. falciparum* density, parasites/µL, %	1.2	1.2	1.2		4.2	10.0	2.4	4.8	2.6	**0.02**

*Demographic, clinical, and parasitologic parameters recorded at enrollment and on day 3 after initiation of artemether/lumefantrine treatment. The study covered 5 districts: Kirundo and Vumbi in Kirundo Province; and Buye, Kiremba, and Ngozi in Ngozi Province. Data are disaggregated by site and province. Continuous variables are reported as medians with IQRs and 95% CIs; categorical variables are expressed as percentages. p values reflect statistical comparisons between the 2 provinces (Kirundo vs. Ngozi); bold indicates significant p values. IQR, interquartile range; –, not applicable. †Day 3 positivity indicates the presence of microscopically detectable asexual *P. falciparum* parasitemia on day 3 after treatment initiation.

Among the 423 children enrolled, 19 (4.5%; 95% CI 2.7%–6.9%) remained parasitemic on day 3 after artemether/lumefantrine treatment. That overall prevalence was below the WHO 10% threshold, but we observed marked heterogeneity across districts. Vumbi (13.6%) exceeded the WHO 10% threshold and neighboring districts, identifying a localized hotspot of delayed clearance. Those differences were statistically significant within Kirundo Province districts (p = 0.0002), but we observed no significant variation between provinces (p = 0.06).

The median asexual parasite density on day 3 was 260 parasites/µL (IQR 141–1,659 parasites/µL), with no significant variation across districts (p = 0.96). Nevertheless, district-specific values showed marked variation, from very low medians in Buye and Kiremba (≈100 parasites/µL) to >1,800 parasites/µL in Ngozi district. Gametocytes were detected microscopically in 11 (2.6%) children on day 3. Their prevalence varied geographically: uncommon in Kirundo Province (1.2%) but more frequent in Ngozi Province (4.8%) and peaked at 10.0% in Kiremba district (Table).

For day 0 isolates, of the 423 children enrolled, 365 (86.3%) samples yielded high-quality sequences and were included in the molecular analysis (Appendix Tables 1, 2). Most (97.5%, 356/365), isolates were wild-type at *pfkelch13*. We detected no validated or candidate WHO-associated mutations linked to ART-R. We did observe nonsynonymous variants in 9 (2.5%) isolates. The most frequent variant was A578S (4 cases, 1.1%), whereas R513S, A626S, H644R, and N672H were each detected in 1 or 2 isolates. None of those polymorphisms are currently associated with delayed parasite clearance, but their detection might indicate ongoing low-level diversification of the *pfkelch13* locus in this setting.

Mutant *pfcrt* haplotypes were nearly fixed. The CVIET triple mutant, defined by mutations at *pfcrt* codons 72–76 (C72/V73/I74/E75/T76), predominated (84.4%, 308/365), whereas the CVIET+356T haplotype was detected in 9.3% (34/365) of isolates. The CVMNK wild-type was uncommon (4.9%, 18/365). Minor variants included SVMNT (72S/76T, 1.1%) and 356T alone (0.3%). We observed no significant difference in overall *pfcrt* haplotype distribution between provinces (p = 0.25). However, intraprovincial heterogeneity was evident, particularly for codon 356 in Ngozi Province (p = 0.01), suggesting microgeographic clustering of resistant variants. The predominance of CVIET confirms that chloroquine resistance remains highly fixed in northern Burundi, with additional mutations such as 356T emerging at low frequency. Of note, we detected no *pfcrt* mutations associated with piperaquine resistance. All isolates were wild-type at positions H97, F145, I218, M343, C350, and G353. Although the 356T variant occurred in 9.6% (35/365) of isolates, that mutation alone does not confer piperaquine resistance.

Mutant haplotypes were common at the *pfmdr1* locus. We identified the wild-type NYSND haplotype, defined by *pfmdr1* codons 86-184-1034-1042-1246 (N86/Y184/S1034/N1042/D1246), in 31.2% (114/365) of isolates. The most frequent mutant was the single 184F allele, detected in 57.3% (209/365) of isolates, followed by 86Y (7.9%). Additional combinations involving 86Y, 1034C, 1042D, or 1246Y remained uncommon (<1.6% each). We noted significant differences in haplotype distribution between provinces (p = 0.03), suggesting that selective pressures on *pfmdr1* might vary geographically, potentially reflecting differences in drug use or drug pressure intensity.

At the *dhfr* locus, resistance alleles were nearly fixed. The triple mutant 51I/59R/108N predominated (92.1%, 336/365), confirming widespread pyrimethamine resistance. Other haplotypes were uncommon, including the double mutant 51I/108N (1.1%) and the single 108N variant (0.5%), and we detected the quadruple mutant 51I/59R/108N/164L in 12 (3.3%) isolates. Although infrequent, the quadruple mutant genotype is clinically relevant because the addition of 164L confers high-grade pyrimethamine resistance and has been associated with sulfadoxine/pyrimethamine treatment failure in East Africa. We detected the wild-type haplotype in only 12 (3.3%) isolates. Variation between provinces was not statistically significant (p = 0.2).

Mutations at the *dhps* locus were also widespread. Only 21/365 (5.8%) isolates were wild-type. The double mutant 437G/540E was detected in 148/365 (40.5%) isolates, whereas the triple mutant 437G/540E/581G was even more frequent (172/365 isolates, 47.1%). Minor haplotypes included 436A (3.8%) and 613S (0.3%). Although we observed no significant difference in overall haplotype proportions between provinces (p = 0.6), district-level variation within Kirundo Province was significant for codons 436A (p = 0.0001), 437G (p = 0.001), and 540E (p = 0.02).

Analysis of combined genotypes enabled inference of multilocus *dhfr*/*dhps* haplotypes, highlighting the extensive accumulation of antifolate resistance alleles. The fully wild-type *dhfr*/*dhps* combination was nearly absent (1/365 isolates, 0.3%). The most prevalent inferred haplotype was the sextuple mutant (*dhfr* triple 51I/59R/108N + *dhps* triple 437G/540E/581G), detected in 161/365 (44.1%) isolates. That genotype has been associated with markedly reduced efficacy of IPTp in East Africa. The classic quintuple mutant (*dhfr* triple 51I/59R/108N + *dhps* double 437G/540E) was also common, found in 134/365 (36.7%) isolates. Together, those 2 high-resistance genotypes accounted for >80% of infections. Less frequent combinations included quadruple or septuple haplotypes, each detected in <3% of isolates. Although overall haplotype distribution did not differ significantly between provinces (p = 0.2), variation occurred at the district level.

We performed molecular analysis on isolates from the 19 children who remained parasitemic on day 3. All isolates were wild-type at *pfkelch13*, confirming the absence of validated or candidate ART-R mutations. At *pfcrt*, the CVIET haplotype predominated (84.2%), and additional variants included CVIET+356T (10.5%) and SVMNT (5.3%). At *pfmdr1*, the 184F allele occurred in 57.9% of isolates, consistent with findings from day 0. Mutations at the *dhfr* locus were nearly fixed, the triple mutant 51I/59R/108N was present in 94.7% of isolates. At *dhps*, the double (437G/540E) and triple (437G/540E/581G) mutants together accounted for >85% of samples. Inferred *dhfr*/*dhps* haplotypes showed a high prevalence of quintuple and sextuple genotypes, mirroring the overall population.

Paired analysis of baseline (day 0) and follow-up (day 3) samples revealed that most isolates retained identical molecular profiles across time points. Nonetheless, we observed a few discordances, particularly in *pfmdr1* (184F/Y) and *dhps* alleles (e.g., detection of 581G or 164L in some day 3 samples). Those differences likely reflect within-host selection of minority clones under drug pressure rather than de novo mutations.

## Discussion

ART-R is a growing threat to malaria control in sub-Saharan Africa, and validated *pfkelch13* mutations are already established in Rwanda, Uganda, and Tanzania (*8*,*9*,*13*,*17*–*20*). Of particular concern is the detection of the *pfkelch13* R561H mutation, a validated marker of delayed parasite clearance, in the Huye district of Rwanda and the Kagera region of Tanzania (*8*–*10*,*12*–*14*,*21*,*22*). Those findings raise the alarm over possible spread into neighboring countries, including Burundi, underscoring the urgent need for localized therapeutic and molecular surveillance.

We adopted a simplified surveillance design to detect ART-R, combining day 3 parasitemia assessment with *pfkelch13* molecular screening. That approach provides complementary clinical and genetic indicators without replacing traditional therapeutic efficacy studies, which remain essential for evaluating ACT efficacy and informing treatment policy. The limited follow-up period and dried blood spot collection enable rapid geographic mapping of ART-R risk across multiple sites in resource-constrained contexts (*23*).

In 5 districts across northern Burundi, 4.5% of children treated with artemether/lumefantrine remained parasitemic on day 3, below the 10% WHO threshold for confirmed ART-R (*24*), but the Vumbi district exceeded the WHO threshold. However, the sample size was calculated for surveillance purposes and was not powered to detect statistically significant differences between districts. Consequently, the geographic comparisons we describe should be interpreted as exploratory findings, rather than definitive evidence of spatial heterogeneity.

None of the day 3 positivity cases harbored validated or candidate *pfkelch13* mutations. That discordance suggests either false-positive results driven by imperfect adherence to the 6-dose artemether/lumefantrine regimen (*25*,*26*), variability in lumefantrine exposure (food intake, vomiting, drug–drug interactions), age-dependent immunity in young children (*27*), or genuine delayed clearance mediated by polygenic architecture of artemisinin response (e.g., *coronin*, *ap2µ*, or *ubp-1*) (*28*,*29*). Pharmacokinetics/adherence verification and broader genomic profiling are needed to resolve those alternatives.

The overall clinical response to artemether/lumefantrine treatment appeared satisfactory despite 4.5% day 3 positivity, likely because lumefantrine, the partner drug with a longer half-life, remains fully effective at eliminating residual parasites. The *pfmdr1* mutations observed (low N86, high 184F) indicate drug pressure but are not validated markers of lumefantrine failure. Those findings suggest lumefantrine efficacy is currently maintaining artemether/lumefantrine effectiveness in Burundi, underscoring the importance of monitoring both artemisinin and partner drug susceptibility.

The absence of *pfkelch13* mutants in Burundi, despite their presence in neighboring countries, might reflect intense transmission (*5*,*7*), where parasite diversity and immunity limit resistant lineage expansion. However, *pfkelch13* mutations have been documented in high-transmission areas of Uganda (*20*), demonstrating that transmission intensity alone does not prevent resistance selection or spread. The absence of validated mutations should not be interpreted as protection. Importation risk remains high, given porous borders and regional connectivity, underscoring the urgency of cross-border surveillance (*24*).

Beyond ART-R, this study revealed a concerning accumulation of antifolate resistance. The *dhfr* triple mutant was nearly fixed, and >80% of infections carried highly resistant multilocus haplotypes. At the *dhps* locus, mutations were also highly prevalent. The 437G/540E/581G triple mutant was present in 47.1% of isolates, whereas only 5.8% of parasites remained wild-type. That pattern indicates widespread sulfadoxine resistance, with direct implications for malaria prevention in Burundi. Sulfadoxine/pyrimethamine is used for IPTp and more recently in PMC. The high prevalence (44%) of the sextuple haplotype raises serious concerns because that genotype is associated with reduced IPTp effectiveness and adverse birth outcomes and likely compromises PMC efficacy. Those findings warrant urgent reassessment of sulfadoxine/pyrimethamine-based prevention strategies in Burundi (*30*–*32*).

ACT partner-drug resistance markers were entrenched. The *pfcrt* CVIET haplotype (74I/75E/76T) predominated (84.4%), with an additional subset carrying 356T (9.3%). Wild-type CVMNK was infrequent (4.9%). Those results confirm that chloroquine resistance remains essentially fixed in the parasite population, consistent with observations across East Africa, despite chloroquine withdrawal (*33*,*34*). That pattern contrasts with observations from several other countries in Africa, where chloroquine-sensitive parasites have reexpanded after drug withdrawal (*35*). The persistence of CVIET in Burundi likely reflects the use of artesunate/amodiaquine as first-line treatment until 2019, which maintained selective pressure favoring *pfcrt* mutant parasites. Similar persistence of CVIET has been reported in other countries that used artesunate/amodiaquine, such as South Sudan. A decline in CVIET prevalence might require several more years to become apparent (*36*–*39*).

We detected no *pfcrt* mutations associated with piperaquine resistance (e.g., F145I, H97Y, M343L, G353V) in this study (*40*–*42*). Those mutations have been identified as key drivers of dihydroartemisinin/piperaquine treatment failures in Southeast Asia, but they appear absent in East Africa parasite populations.

At the *pfmdr1* locus, the Y184F mutation was the dominant variant (57.3%), whereas the wild-type NYSND was found in 31.2%. Other variants involving 86Y, 1034C, 1042D, or 1246Y were uncommon (<1.6%). The predominance of 184F is consistent with reports from Zanzibar (Tanzania) and Senegal (*43*,*44*). The Y184F mutation is frequently selected by artemether/lumefantrine treatment and can modulate parasite responses in vitro, but it should be regarded as a marker of drug pressure rather than a validated clinical marker of lumefantrine resistance (*45*).

The limitations of this study include partially supervised drug treatment, unmeasured drug concentrations, day 3 follow-up only, and no assessment of copy number variation in *pfmdr1* and *plasmepsin 2/3*, the established markers associated with reduced lumefantrine (*46*–*48*) and piperaquine (*40*,*49*,*50*) susceptibility. Study strengths include multiple sentinel sites in a high-risk border region, combined clinical and molecular surveillance, and an integrated baseline dataset informing national and regional strategies.

WHO recommends deploying multiple first-line ACTs to delay malaria drug resistance emergence. In Burundi, where artemether/lumefantrine is the sole first-line treatment, introducing an alternative such as dihydroartemisinin/piperaquine could reduce selective pressure. The absence of *pfcrt* mutations associated with piperaquine resistance suggests dihydroartemisinin/piperaquine could retain full efficacy and serve as a second first-line option.

In conclusion, our findings highlight a critical juncture for Burundi. We detected no *pfkelch13* mutants, but excess localized day 3 positivity and entrenched sulfadoxine/pyrimethamine resistance represent clear warning signals. High-transmission dynamics may currently protect Burundi against *pfkelch13*-mediated ART-R, but that protection is fragile. Strengthened therapeutic efficacy monitoring, expanded molecular surveillance, including genomic approaches, and cross-border collaboration are urgently required to prevent resistant *P. falciparum* establishment and spread in the Great Lakes region of Burundi.

Appendix Additional information for border region surveillance of malaria drug resistance, northern Burundi, 2023–2024.
