# Supplementary material for: Border Region Surveillance of Malaria Drug Resistance, Northern Burundi, 2023–2024
Source: Emerg Infect Dis. 2026 Apr;32(4):553–62. doi: 10.3201/eid3204.251711 (PMC13094827; doi:10.3201/eid3204.251711)
Supplement: Appendix — Additional information for border region surveillance of malaria drug resistance, northern Burundi, 2023–2024. [file 25-1711-Techapp-s1.pdf]

Article DOI: https://doi.org/10.3201/eid3204.251711

EID cannot ensure accessibility for supplementary materials supplied by authors. Readers who have difficulty accessing supplementary content should contact the authors for assistance.

Border Region Surveillance of Malaria Drug Resistance, Northern Burundi, 2023–2024

Appendix

Appendix Table 1. Distribution of *Plasmodium falciparum* resistance-associated mutations across eight sentinel sites in northern Burundi*

			KIRUNDO			NGOZI					
Gene	Codon	AA	KIRUNDO	VUMBI	Total	BUYE	KIREMBA	NGOZI	Total	Total	*p-value*
No. samples			155	62	217	76	30	42	148	**365**	
Pfkelch13	WT	-	149 (96.1)	62 (100)	211 (97.2)	74 (97.4)	29 (96.7)	42 (100)	145 (98.0)	**356 (97.5)**	0.5
	513	R>S	1 (0.6)	0	1 (0.5)	0	0	0	0	**1 (0.3)**	
	578	A>S	2 (1.3)	0	2 (0.9)	1 (1.3)	1 (3.3)	0	2 (1.4)	**4 (1.1)**	
	626	A>S	1 (0.6)	0	1 (0.5)	0	0	0	0	**1 (0.3)**	
	644	H>R	0	0	0	1 (1.3)	0	0	1 (0.7)	**1 (0.3)**	
	672	N>H	2 (1.3)	0	2 (0.9)	0	0	0	0	**2 (0.5)**	
Pfcrt	72	C	155 (100)	61 (98.4)	216 (99.5)	73 (96.1)	30 (100)	42 (100)	145 (98.0)	**361 (98.9)**	0.16
		S	0	1 (1.6)	1 (0.5)	3 (3.9)	0	0	3 (2.0)	**4 (1.1)**	
	74	M	8 (5.2)	5 (8.1)	13 (6.0)	1 (1.3)	1 (3.3)	4 (9.5)	**6 (4.1)**	**19 (5.2)**	0.4
		I	147 (94.8)	57 (91.9)	204 (94.0)	75 (98.7)	29 (96.7)	38 (90.5)	**142 (95.9)**	**346 (94.8)**	
	75	N	8 (5.2)	5 (8.1)	13 (6.0)	1 (1.3)	1 (3.3)	4 (9.5)	**6 (4.1)**	**19 (5.2)**	0.4
		E	147 (94.8)	57 (91.9)	204 (94.0)	75 (98.7)	29 (96.7)	38 (90.5)	**142 (95.9)**	**346 (94.8)**	
	76	K	8 (5.2)	5 (8.1)	13 (6.0)	1 (1.3)	1 (3.3)	4 (9.5)	**6 (4.1)**	**19 (5.2)**	0.4
		T	147 (94.8)	57 (91.9)	204 (94.0)	75 (98.7)	29 (96.7)	38 (90.5)	**142 (95.9)**	**346 (94.8)**	
	93	T	155 (100)	62 (100)	217 (100)	76 (100)	30 (100)	42 (100)	148 (100	**365 (100)**	-
		S	0	0	0	0	0	0	0	**0**	
	97	H	155 (100)	62 (100)	217 (100)	76 (100)	30 (100)	42 (100)	148 (100	**365 (100)**	-
		Y	0	0	0	0	0	0	0	**0**	
	145	F	155 (100)	62 (100)	217 (100)	76 (100)	30 (100)	42 (100)	148 (100	**365 (100)**	-
		I	0	0	0	0	0	0	0	**0**	
	218	I	155 (100)	62 (100)	217 (100)	76 (100)	30 (100)	42 (100)	148 (100	**365 (100)**	-
		F	0	0	0	0	0	0	0	**0**	
	343	M	155 (100)	62 (100)	217 (100)	76 (100)	30 (100)	42 (100)	148 (100	**365 (100)**	-
		L	0	0	0	0	0	0	0	**0**	
	350	C	155 (100)	62 (100)	217 (100)	76 (100)	30 (100)	42 (100)	148 (100	**365 (100)**	-
		R	0	0	0	0	0	0	0	**0**	
	353	G	155 (100)	62 (100)	217 (100)	76 (100)	30 (100)	42 (100)	148 (100	**365 (100)**	-

			KIRUNDO			NGOZI					
Gene	Codon	AA	KIRUNDO	VUMBI	Total	BUYE	KIREMBA	NGOZI	Total	Total	*p-value*
		V	0	0	0	0	0	0	0	**0**	
	356	I	142 (91.6)	57 (91.9)	199 (91.7)	71 (93.4)	28 (93.3)	32 (76.2)	131 (88.5)	330 (90.4)	0.31
		T	13 (8.4)	5 (8.1)	18 (8.3)	5 (6.6)	2 (6.7)	10 (23.8)	17 (11.5)	35 (9.6)	
Pfmdr1	86	N	137 (88.4)	52 (83.9)	189 (87.1)	73 (96.1)	29 (96.7)	38 (90.5)	140 (94.6)	**329 (90.1)**	**0.02**
		Y	18 (11.6)	10 (16.1)	28 (12.9)	3 (3.9)	1 (3.3)	4 (9.5)	8 (5.4)	**36 (9.9)**	
	184	Y	58 (37.4)	30 (48.4)	88 (40.6)	23 (30.3)	12 (40.0)	21 (50.0)	56 (37.8)	**144 (39.5)**	0.6
		F	97 (62.6)	32 (51.6)	129 (59.4)	53 (69.7)	18 (60.0)	21 (50.0)	92 (62.2)	**221 (60.5)**	
	1034	S	153 (98.7)	60 (96.8)	213 (98.2)	74 (97.4)	30 (100)	41 (97.6)	145 (98.0)	**358 (98.1)**	0.9
		C	2 (1.3)	2 (3.2)	4 (1.8)	2 (2.6)	0	1 (2.4)	3 (2.0)	**7 (1.9)**	
	1042	N	153 (98.7)	61 (98.4)	214 (98.6)	74 (97.4)	30 (100)	41 (97.6)	145 (98.0)	**359 (98.4)**	0.6
		D	2 (1.3)	1 (1.6)	3 (1.4)	2 (2.6)	0	1 (2.4)	3 (2.0)	**6 (1.6)**	
	1246	D	155 (100)	62 (100)	217 (100)	74 (97.4)	30 (100)	41 (97.6)	145 (98.0)	**362 (99.2)**	**0.03**
		Y	0	0	0	2 (2.6)	0	1 (2.4)	3 (2.0)	**3 (0.8)**	
dhfr	16	A	155 (100)	62 (100)	217 (100)	76 (100)	30 (100)	42 (100)	148 (100)	**365 (100)**	-
		V	0	0	0	0	0	0	0	**0**	
	50	C	155 (100)	62 (100)	217 (100)	76 (100)	30 (100)	42 (100)	148 (100)	**365 (100)**	-
		R	0	0	0	0	0	0	0	**0**	
	51	N	10 (6.5)	2 (3.2)	13 (6.0)	1 (1.3)	0	0	1 (0.7)	**14 (3.8)**	**0.01**
		I	145 (93.5)	59 (95.2)	204 (94.0)	75 (98.7)	30 (100)	42 (100)	147 (99.3)	**351 (96.2)**	
	59	C	10 (6.5)	4 (6.5)	14 (6.5)	1 (1.3)	1 (3.3)	2 (4.8)	4 (2.7)	**18 (4.9)**	0.1
		R	145 (93.5)	58 (93.5)	203 (93.5)	75 (98.7)	29 (96.7)	40 (95.2)	144 (97.3)	**347 (95.1)**	
	108	S	8 (5.2)	3 (4.8)	11 (5.1)	1 (1.3)	0	0	1 (0.7)	**12 (3.3)**	0.02
		N	147 (94.8)	59 (95.2)	206 (94.9)	75 (98.7)	30 (100)	42 (100)	147 (99.3)	**353 (96.7)**	
	164	I	150 (97.0)	61 (98.4)	211 (97.2)	72 (94.7)	30 (100)	41 (97.6)	143 (96.6)	**354 (97.0)**	0.7
		L	5 (3.2)	1 (1.6)	6 (2.8)	4 (5.3)	0	1 (2.4)	5 (3.4)	**11 (3.0)**	
dhps	431	I	155 (100)	62 (100)	217 (100)	76 (100)	30 (100)	42 (100)	148 (100)	**365 (100)**	-
		V	0	0	0	0	0	0	0	**0**	
	436	S	153 (98.7)	53 (85.5)	206 (94.9)	76 (100)	29 (96.7)	40 (95.2)	145 (98.0)	**351 (96.2)**	0.1
		A	2 (1.3)	9 (14.5)	11 (5.1)	0	1 (3.3)	2 (4.8)	3 (2.0)	**14 (3.8)**	
	437	A	9 (5.8)	10 (16.1)	19 (8.8)	4 (5.3)	1 (3.3)	6 (14.3)	11 (7.4)	**30 (8.2)**	0.6
		G	146 (94.2)	52 (83.9)	198 (91.2)	72 (94.7)	29 (96.7)	36 (85.7)	137 (92.6)	**335 (91.8)**	
	540	K	5 (3.2)	7 (11.3)	14 (6.5)	4 (5.3)	1 (3.3)	4 (9.5)	9 (6.1)	**21 (5.8)**	0.8
		E	150 (96.8)	55 (88.7)	203 (93.5)	72 (94.7)	29 (96.7)	38 (90.5)	139 (93.9)	**344 (94.2)**	
	581	A	76 (49.0)	31 (50.0)	107 (49.3)	37 (48.7)	14 (46.7)	23 (54.8)	74 (50.0)	**181 (49.6)**	0.9
		G	79 (51.0)	51 (50.0)	110 (50.7)	39 (51.3)	16 (53.3)	19 (45.2)	74 (50.0)	**184 (50.4)**	
	613	A	154 (99.4)	62 (100)	216 (99.5)	76 (100)	30 (100)	42 (100)	148 (100)	**364 (99.7)**	0.4
		S	1 (0.6)	0	1 (0.5)	0	0	0	0	**1 (0.3)**	

*The table presents the frequency of amino acid variants in *pfkelch13*, *pfcrt*, *pfmdr1*, *dhfr*, and *dhps* genes across individual sites grouped by province (Kirundo and Ngozi), and for the entire dataset (N = 365). Mutant and wild-type alleles are listed for each codon. Site-level and overall p-values (Chi-square or Fisher's exact test, as appropriate) compare allele frequencies between health centers and provinces. P-values show difference in proportions between provinces. Significant p-values ($p < 0.05$) are shown in bold. Total percentages are calculated relative to the number of samples successfully genotyped per locus.

Appendix Table 2. Distribution of single nucleotide polymorphisms (SNPs) and haplotypes in *pfkelch13*, *pfcrt*, *pfmdr1*, *dhfr*, and *dhps* genes across eight sentinel sites in northern Burundi*

			KIRUNDO			NGOZI					p-
**Gene**	**Allele**		**KIRUNDO**	**VUMBI**	**Total**	**BUYE**	**KIREMBA**	**NGOZI**	**Total**	**Total**	**value**
No. of sample (%)			155	62	217	76	30	42	148	365	-
Pfkelch13	WT	-	149 (96.1)	62 (100)	211 (97.2)	74 (97.4)	29 (96.7)	42 (100)	145 (98.0)	356 (97.5)	0.5
	513	R>S	1 (0.6)	0	1 (0.5)	0	0	0	0	1 (0.3)	
	578	A>S	2 (1.3)	0	2 (0.9)	1 (1.3)	1 (3.3)	0	2 (1.4)	4 (1.1)	
	626	A>S	1 (0.6)	0	1 (0.5)	0	0	0	0	1 (0.3)	
	644	H>R	0	0	0	1 (1.3)	0	0	1 (0.7)	1 (0.3)	
	672	N>H	2 (1.3)	0	2 (0.9)	0	0	0	0	2 (0.5)	
Pfcrt	WT	CVMNKTHFIMCGI	8 (5.2)	5 (8.1)	13 (6.0)	1 (1.3)	1 (3.3)	3 (7.1)	5 (3.4)	18 (4.9)	0.25
	72S/76T	SVMNTTHFIMCGI	0	1 (1.6)	1 (0.5)	3 (3.9)	0	0	3 (2.0)	4 (1.1)	
	74I/75E/76T	CVIETTHFIMCGI	134 (69.0)	51 (82.3)	185 (85.3)	67 (88.2)	27 (90.0)	29 (82.3)	123 (83.1)	308 (84.4)	
	356T	CVMNKTHFIMCGT	0	0	0	0	0	1 (2.4)	1 (0.7)	1 (0.3)	
	74I/75E/76T/356T	CVIETTHFIMCGT	13 (8.4)	5 (8.1)	18 (8.3)	5 (6.6)	2 (6.7)	9 (21.4)	16 (10.8)	34 (9.3)	
Pfmdr1	WT	NYSND	43 (27.7)	23 (37.1)	66 (30.4)	20 (26.3)	11 (36.7)	17 (40.5)	48 (32.4)	114 (31.2)	**0.03**
	86Y	YYSND	15 (9.7)	6 (9.7)	21 (9.7)	3 (3.9)	1 (3.3)	4 (9.5)	8 (5.4)	29 (7.9)	
	184F	NFSND	92 (59.4)	28 (45.2)	120 (55.3)	51 (67.1)	18 (60.0)	20 (47.6)	89 (60.1)	209 (57.3)	
	86Y/184F	YFSND	3 (1.9)	3 (4.8)	6 (2.8)	0	0	0	0	6 (1.6)	
	86Y/1034C	YYCND	0	1 (1.6)	1 (0.5)	0	0	0	0	1 (0.3)	
	184F/1034C/1042D	NFCDD	2 (1.3)	1 (1.6)	3 (1.4)	0	0	0	0	3 (0.8)	
	184F/1034C/1042D/1246Y	NFCDY	0	0	0	2 (2.6)	0	1 (2.4)	3 (2.0)	3 (0.8)	
dhfr	WT	ACNCSI	8 (5.2)	3 (4.8)	11 (5.1)	1 (1.3)	0	0	1 (0.7)	12 (3.3)	0.2
	108N	ACNCNI	2 (1.3)	0	2 (0.9)	0	0	0	0	2 (0.5)	
	51I/108N	ACICNI	0	1 (1.6)	1 (0.5)	0	1 (3.3)	2 (4.8)	3 (2.0)	4 (1.1)	
	51I/59R/108N	ACIRNI	140 (90.3)	57 (91.9)	197 (90.8)	71 (93.4)	29 (96.7)	39 (92.9)	139 (93.9)	336 (92.1)	
	51I/59R/108N/164L	ACIRNL	5 (5.2)	1 (1.6)	11 (5.1)	4 (5.3)	0	1 (2.4)	1 (0.7)	12 (3.3)	
dhps	WT	ISAKAA	5 (3.2)	7 (11.3)	12 (5.5)	4 (5.3)	1 (3.3)	4 (9.5)	9 (6.1)	21 (5.8)	0.6
	540E	ISAEAA	4 (2.6)	3 (4.8)	7 (3.2)	0	0	2 (4.8)	2 (1.4)	9 (2.5)	
	437G/540E	ISGEAA	67 (43.2)	19 (30.6)	86 (39.6)	33 (43.4)	13 (43.3)	16 (38.1)	62 (41.9)	148 (40.5)	
	436A/437G/540E	IAGEAA	0	2 (3.2)	2 (0.8)	0	0	1 (2.4)	1 (0.7)	3 (0.8)	
	437G/540E/581G	ISGEGA	76 (49.0)	24 (38.7)	100 (46.1)	39 (51.3)	15 (50.0)	18 (42.9)	72 (48.6)	172 (47.1)	
	436A/437G/540E/581G	IAGEGA	2 (1.3)	7 (11.3)	9 (4.1)	0	1 (3.3)	1 (2.4)	2 (1.4)	11 (3.0)	
	437G/540E/581G/613S	ISGEGS	1 (0.6)	0	1 (0.5)	0	0	0	0	1 (0.3)	

Gene	Allele		KIRUNDO			NGOZI					p-value
			KIRUNDO	VUMBI	Total	BUYE	KIREMBA	NGOZI	Total	Total	
dhfr_dhps	Wild-type	WT_WT	0	0	0	1 (1.3)	0	0	1 (0.7)	1 (0.3%)	0.2
	Double mutant	51IR/108N_WT	0	1 (1.6)	1 (0.5)	0	0	0	0	1 (0.3)	
		WT_437G/540E	5 (3.2)	1 (1.6)	6 (2.8)	0	0	0	0	6 (1.6)	
	Triple mutant	WT_437G/540E/581G	3 (1.9)	1 (1.6)	4 (1.8)	0	0	0	0	4 (1.1)	
		51I/108N_540E	0	0	0	0	0	1 (2.4)	1 (0.7)	1 (0.3%)	
		51I/59R/108N_WT	5 (3.2)	6 (9.7)	11 (5.1)	3 (3.9)	1 (3.3)	4 (9.5)	8 (5.4)	19 (5.2)	
		108N_437G/540E	1 (0.6)	0	1 (0.5)	0	0	0	0	1 (0.3%)	
	Quadruple mutant	108N_437G/540E/581G	1 (0.6)	0	1 (0.5)	0	0		0	1 (0.3%)	
		51I/59R/108N_540E	4 (2.6)	3 (4.8)	7 (3.2)	0	0	1 (2.4)	1 (0.7)	8 (2.2)	
		WT_436A/437G/540E/581G	0	1 (1.6)	1 (0.5)	0	0	0	0	1 (0.3%)	
	Quintuple mutant	51I/108N_437G/540E/581G	0	0	0	0	1 (3.3)	1 (2.4)	2 (1.4)	2 (0.5)	
		51I/59R/108N_437G/540E	58 (37.4)	17 (27.4)	75 (34.6)	30 (39.5)	13 (43.3)	16 (38.1)	59 (39.9)	134 (36.7)	
	Sextuple mutant	51I/59R/108N_436A/437G/540E	0	2 (3.2)	2 (0.9)	0	0	1 (2.4)	1 (0.7)	3 (0.8)	
		51I/59R/108N_437G/540E/581G	70 (45.2)	23 (37.1)	93 (42.9)	38 (50.0)	14 (46.7)	16 (38.1)	68 (45.9)	161 (44.1)	
		51I/59R/108N/164L_437G/540E	3 (1.9)	1 (1.6)	4 (1.8)	3 (3.9)	0	0	3 (2.0)	7 (1.9)	
	Septuple mutant	51I/59R/108N_436A/437G/540E/581G	2 (1.3)	6 (9.7)	8 (3.7)	0	1 (3.3)	1 (2.4)	2 (1.4)	10 (2.7)	
		51I/59R/108N_437G/540E/581G/613S	1 (0.6)	0	1 (0.5)	0	0	0	0	1 (0.3)	
		51I/59R/108N/164L_437G/540E/581G	2 (1.3)	0	2 (0.9)	1 (1.3)	0	1 (2.4)	2 (0.9)	4 (1.1)	

*The table shows the frequency of point mutations and derived haplotypes conferring resistance to artemisinin and partner drugs in *Plasmodium falciparum*, grouped by province (Kirundo and Ngozi). Wild-type and mutant alleles are indicated for each codon or haplotype. Combined *dhfr/dhps* haplotypes are classified as wild-type, single, double, triple, quadruple, quintuple, or sextuple mutants. Percentages were calculated among successfully genotyped samples for each gene. p-values were derived from Chi-square or Fisher's exact test as appropriate. P-values show difference in proportions between provinces. Statistically significant p-values ($p < 0.05$) are shown in bold.
